# New Auroras on the Roles of the Chromosomal Passenger Complex in Cytokinesis: Implications for Cancer Therapies

**DOI:** 10.3389/fonc.2015.00221

**Published:** 2015-10-14

**Authors:** Pier Paolo D’Avino, Luisa Capalbo

**Affiliations:** ^1^Department of Pathology, University of Cambridge, Cambridge, UK

**Keywords:** cell division, microtubule, Aurora B, abscission, anticancer therapies

## Abstract

The chromosomal passenger complex (CPC), composed of a kinase component, Aurora B, the scaffolding subunit inner centromeric protein, Borealin, and Survivin, is a key regulator of cell division. It controls multiple events, from chromosome condensation in prophase to the final separation or abscission of the two daughter cells. The essential functions of the CPC during metaphase, however, have always hindered an accurate study of its role during cytokinesis. The recent development of small molecule inhibitors against Aurora B and the use of elegant technologies such as chemical genetics have offered new approaches to study the functions of the CPC at the end of cell division. Here, we review the recent findings about the roles of the CPC in controlling the assembly of the cleavage furrow, central spindle, and midbody. We will also discuss the crucial function of this complex in controlling abscission timing in order to prevent abscission when lagging chromatin is present at the cleavage site, thereby avoiding the formation of genetically abnormal daughter cells. Finally, we offer our perspective on how to exploit the potential therapeutic applications of inhibiting CPC activity during cytokinesis in cancer cells.

## Introduction

Faithful chromosome segregation during cell division is crucial for growth, development, and reproduction in many organisms. Defects in this process have been associated with various genetic diseases, including cancer. For example, many cancer cells present chromosomal instability (CIN), which contributes to carcinogenesis by altering the balance of critical growth and death pathways and the overall expression of oncogenes and tumor suppressors. Thus, understanding the mechanisms that control genome segregation during mitosis can reveal some of the processes that promote genomic instability in cancer. Consistent with this, animal models have shown that failure in controlling either chromosome segregation or the final separation of the two dividing cells – cytokinesis – can cause CIN and carcinogenesis (1–4). Moreover, one of the hallmarks of cancer is uncontrolled cell proliferation, and many cell division regulators are validated targets for the isolation of novel chemotherapeutic drugs for the treatment of cancer pathologies. In particular, mitotic serine/threonine kinases have become an intensively studied class of anticancer drug targets, and inhibitors of mitotic kinases, such as Aurora and Polo-like kinases, are currently undergoing clinical trials (5). A comprehensive knowledge of the function of these kinases is therefore crucial to identify new pathways and biomarkers that could aid in the design of better-targeted and less toxic anticancer therapies.

**Figure 1:**
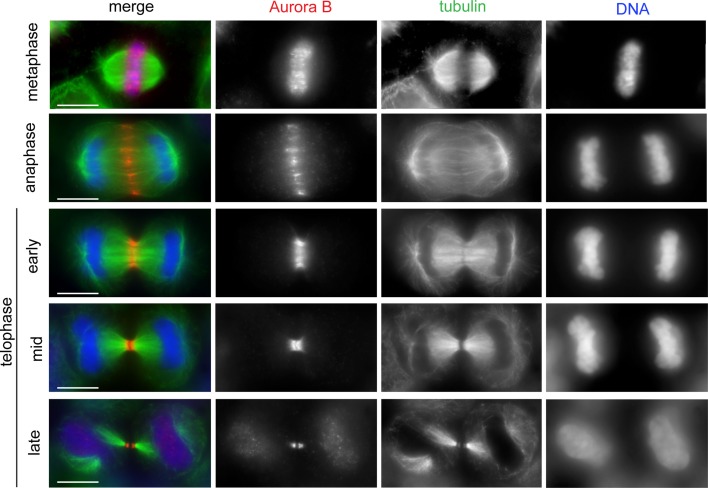
**The CPC shows dynamic localization during mitosis and cytokinesis**. HeLa cells were fixed and stained to reveal Aurora B (red), tubulin (green), and DNA (blue). The CPC (here represented by Aurora B) translocated from the mitotic chromosomes to the central spindle early in anaphase. In early telophase, the CPC accumulated at the midbody arms. Scale bars: 10 μm.

**Figure 2:**
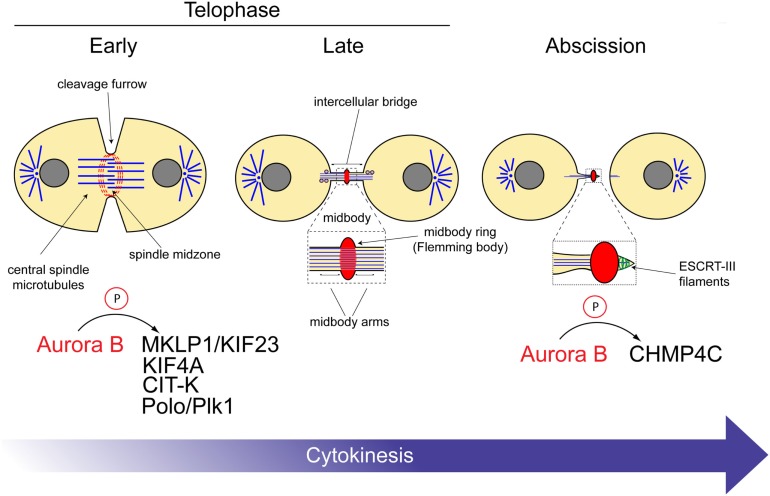
**Aurora B substrates during cytokinesis**. Schematic diagram illustrating the roles of the CPC at different stages of cytokinesis. The substrates of Aurora B are indicated at the bottom. See text for details.

The chromosomal passenger complex (CPC) is one of the major regulators of cell division in all eukaryotes and is composed of four subunits: the scaffolding component inner centromeric protein (INCENP), Borealin, Survivin, and Aurora B kinase [reviewed by Carmena et al. (6)]. The name of the complex reflects its dynamic distribution during mitosis. It localizes to centromeres until chromosome segregation and then relocates to the central spindle, an array of antiparallel and interdigitating microtubules that forms between the separating sister chromatids after anaphase onset (Figure 1). The translocation from centromeres to the central spindle depends on the interaction of INCENP’s coiled-coiled domain with microtubules and requires the kinesin MKLP2/KIF20A (7, 8). Consistent with this localization, the CPC controls various events throughout cell division, from chromosome condensation in prophase to the final separation or abscission of the two daughter cells. The best-known and most studied role of the CPC is undoubtedly the correction of improper kinetochore–microtubule attachments in prometaphase, but there is a growing evidence that the CPC plays important roles also during cytokinesis. Cytokinesis is mediated by the constriction of an actomyosin contractile ring at the equatorial cell cortex that bisects the dividing cell. As mentioned earlier, after anaphase onset, microtubules reassemble to form the central spindle, an array of antiparallel microtubules that overlap at their plus ends in a region called the spindle midzone (Figure 2). Central spindle and astral microtubules cooperate to activate the small GTPase RhoA at the equatorial cortex, which in turn triggers the assembly and constriction of an actomyosin ring responsible for cleavage furrow ingression. Furrow ingression progressively compacts the central spindle to form an organelle known as the midbody, which provides a platform important for the recruitment and organization of many proteins that regulate the final abscission of the two daughter cells (Figure 2). Here, we review recent findings about the role of the CPC in controlling cleavage furrow ingression, the formation and dynamics of the central spindle, the architecture of the midbody, and abscission. Finally, we offer our perspective on the possibility of exploiting the roles of the CPC in cytokinesis for anticancer therapy.

## The CPC Promotes Central Spindle and Midbody Formation

Various microtubule-associated proteins cooperate to regulate the assembly and dynamics of central spindle microtubules [reviewed by Douglas and Mishima (9)]. The CPC has been shown to control the activities of at least two central spindle kinesin motors, MKLP1/KIF23 and KIF4A (Figure 2).

MKLP1 is the motor component of the centralspindlin complex, a heterotetramer composed of two MKLP1 subunits and two molecules of RacGAP1/MgcRacGAP/Cyk4 (10). Centralspindlin is required for central spindle formation in many organisms, from nematodes to humans (10–13), but is also known to perform many other crucial roles during cytokinesis. For example, the RacGAP1 subunit interacts with and activates the RhoGEF Ect2, which in turn promotes RhoA activation and contractile ring assembly and constriction (14–17). RacGAP1 also associates with the plasma membrane through its C1 domain, and this interaction provides a crucial link between the midbody and the membrane, important for the final abscission of the two daughter cells (18). Centralspindlin activity is also essential for microtubule bundling (19) and formation of the central spindle and midbody. To exert these functions, centralspindlin complexes need to oligomerize, and this clustering is promoted by Aurora B phosphorylation of the serine 708 in the MKLP1 C-terminal tail. This phosphorylation prevents the association of MKLP1 with 14-3-3 protein, which inhibits centralspindlin clustering (20). Thus, Aurora B promotes the assembly of the central spindle via phosphorylation of the kinesin component of the centralspindlin complex.

The kinesin KIF4A and the microtubule-associated protein PRC1 form another complex important for central spindle assembly. PRC1 is able to cross-link and bundle microtubules and is transported to the spindle midzone by KIF4A (21–25). The formation of the PRC1/KIF4A complex is prevented by cyclin-dependent kinase 1 (Cdk1) phosphorylation in metaphase (24), but after anaphase onset, this interaction is instead promoted through Aurora B phosphorylation of KIF4A (26). This phosphorylation also stimulates the microtubule-dependent ATPase activity of KIF4A, which suppresses microtubule dynamics and limits the length of the central spindle (26). Quite interestingly, KIF4A is also responsible for maintaining phosphatase PP2A-B56 at the central spindle, thereby creating a spatially restricted negative feedback loop counteracting Aurora B in cytokinesis (27).

We have recently found that the CPC directly interacts with citron kinase (CIT-K), an important midbody protein that links a network of contractile ring and central spindle proteins, including actin, anillin, myosin, MKLP1, KIF14, and RhoA, in both *Drosophila* and human cells (28–32). The CPC and CIT-K depend on each other for proper localization to the midbody and Aurora B phosphorylates CIT-K to control its localization and interaction with central spindle partners (McKenzie et al., submitted). Thus, a cross-regulatory mechanism between two important kinases seems to regulate proper midbody architecture and successful completion of cytokinesis.

New evidence also involved Aurora B in the regulation of Polo kinase during cytokinesis in *Drosophila*. Polo kinase was the first identified member of the evolutionary conserved family of Polo-like kinases (Plk). All of the members of this family have essential roles during cell division (33). Aurora B phosphorylates Polo on its activation loop to promote its kinase activity in mitosis (34). In cytokinesis, Aurora B-mediated phosphorylation of Polo is responsible for its translocation from central spindle microtubules to the midbody. Failure in this process induces cytokinesis defects (35). Not much is known about Polo function at the end of cytokinesis, but its localization is probably necessary to activate substrates essential for abscission.

In conclusion, together these data indicate that the CPC orchestrates the activity of various proteins to regulate the correct assembly and size of both the central spindle and midbody (Figure 2).

## Is There a Role for the CPC in Cleavage Plane Positioning?

The CPC was found to accumulate at the spindle midzone and equatorial cortex very rapidly after anaphase onset (Figure 1) (36), leading to the proposal that this localization could reflect a role for the CPC in positioning the division site and promoting contractile ring assembly. In 2008, a study reported that CPC translocation to the central spindle generates an Aurora B phosphorylation gradient that has its peak at the spindle midzone (37). This Aurora B gradient has been suggested to determine the position of the cleavage plane, but the molecular mechanisms are still lacking. More recently, another group has reported that Aurora B-mediated centralspindlin clustering is important to promote the interaction of the RacGAP1 with the plasma membrane and proposed that this event could promote RhoA activation at the cleavage furrow in both nematodes and human cells (38). In conclusion, although these data point to a potential role for Aurora B in furrow formation, further studies are needed to define the molecules and mechanisms involved in this process.

## The CPC Regulates Abscission Timing

The CPC has been proposed to prevent abscission in the presence of DNA at the cleavage site, thereby avoiding the formation of genetically abnormal daughter cells (39). In this study, it was reported that if lagging chromatin lingered at the cleavage site, Aurora B remained active and stabilized the intercellular bridge. The Aurora B target(s) in abscission, however, have remained elusive until a few years ago when two studies simultaneously showed that one of such targets is the Snf7 component of the endosomal sorting complex required for transport III (ESCRT-III) (40, 41). ESCRT proteins are evolutionarily conserved and best known for catalyzing membrane fission events both in virus budding and in the sorting of receptors into vesicles that bud off into the lumen of the endosome, creating multivesicular bodies (MVBs) (42). The ESCRT-III complex provides the core machinery that mediates membrane deformation and fission events during these events (43) as well as during abscission, which is topologically similar to MVB biogenesis and virus budding (44). Consistent with this, the ESCRT-III Snf7 components (known as CHMP4 proteins in humans) form spiral filaments that appear to remodel and constrict the membrane in order to create the abscission site (45). The CPC has been proposed to regulate abscission timing through direct interaction of the ESCRT-III Snf7 components both in *Drosophila* and humans (40, 41). In human cells, Borealin directly interacts with all three CHMP4 proteins, CHMP4A, CHMP4B, and CHMP4C, and Aurora B phosphorylates the terminal tail of CHMP4C. Two different models have been proposed to explain the regulation of CHMP4 proteins by the CPC. Carlton et al. (41) proposed that Aurora B phosphorylation promotes CHMP4C translocation to the midbody ring, where this ESCRT-III component inhibits abscission. By contrast, we proposed that CPC controls abscission through inhibition of CHMP4 polymerization and membrane association using two concurrent mechanisms: interaction of its Borealin component with all three CHMP4 proteins and phosphorylation of CHMP4C by Aurora B (40). These two concomitant events could preclude the formation of the ESCRT-III filaments essential for the formation of the constriction that physically separate the two daughter cells. In this model, CHMP4 proteins could assemble into spiral filaments only after CPC removal from the midbody. Overall, the CPC-mediated regulation of ESCRT-III has been suggested to act as a surveillance mechanism that prevents abscission in the presence of DNA at the cleavage site (39–41) (Figure 2).

## Targeting CPC Functions in Cytokinesis: An Alternative to the Use of Aurora B Small Molecule Inhibitors in Cancer Therapy?

Aurora kinases are overexpressed and amplified in many tumors, and Aurora A, but not Aurora B, displays oncogenic properties (46–48). However, polyploid cells overexpressing Aurora B can induce tumor formation when injected in nude mice, indicating that high levels of this kinase can be tumorigenic when coupled with cytokinesis failure (49). Consistent with this, tetraploid cells are more sensitive to Aurora B inhibition (50). Moreover, overexpression of Aurora B has been correlated with poor prognosis in a large number of cancers, including breast, ovarian, lung, nasopharyngeal, and hepatocellular carcinomas (51–55). This evidence has led to the development of small molecule inhibitors designed to interfere with the ATP-binding pocket of Aurora kinases that are currently in clinical trials for the treatment of various cancer pathologies (5, 56). Aurora B inhibitors have the ability to silence the spindle assembly checkpoint causing premature mitotic exit and consequent chromosome mis-segregation, cytokinesis failure, and nuclear fragmentation. All these defects ultimately lead to cell death, and this antiproliferative effect could potentially affect cancer cells that rely on Aurora B overexpression more than normal cells. However, in the long term, Aurora B inhibitors also interfere with the division of normal cells and indeed clinical toxicity profiles of Aurora inhibitors indicated frequent side effects such as myelosuppression, febrile neutropenia, and gastrointestinal problems (nausea, diarrhea, and mucositis), some of which have been directly attributed to Aurora B inhibition (5, 56). Furthermore, ATP-binding competitors are often not very selective and can inhibit the activity of other kinases. Thus, there is a need to develop alternative, more selective, and less toxic approaches to inhibit Aurora B activity.

There is evidence that targeting mitotic exit without perturbing spindle assembly could potentially be a more effective cancer treatment (57). In addition, low levels of cytokinesis failure do not seem to affect the shape and size of proliferative tissues in invertebrate animal models. For example, actively proliferating tissues such as brains and imaginal disks (i.e., the tissues that give rise to the adult fly) of larvae carrying strong mutant combinations of the *Drosophila* CIT-K homologue are highly polyploid (8N or more), misshapen, and smaller than their wild-type counterparts. By contrast, the same tissues of larvae carrying weaker allelic combinations are mostly tetraploid and normal in shape and size (58). These results indicate that, at least in *Drosophila*, organs can tolerate the presence of a considerable number of tetraploid cells and thus one single event of cytokinesis failure does not appear to significantly interfere with tissue development and function, whereas multiple cytokinesis failures lead to cell death and impair tissue development. Therefore, it is conceivable that inhibition of cytokinesis could selectively affect the proliferation of very actively dividing cells. There is also another motive to hypothesize that cytokinesis failure could selectively eliminate cancer cells. It is well established that many carcinomas present numerical chromosomal abnormalities (aneuploidy and/or polyploidy) and instability (59, 60). Therefore, provoking cytokinesis failure in these already chromosomally abnormal cancer cells could very rapidly increase their genomic content above a threshold compatible with cell viability. Together, these data indicate that targeting the cytokinetic functions of the CPC could be a valid alternative strategy for antiproliferative cancer therapy. Clearly, inhibitors of Aurora B kinase activity cannot be used to impair CPC functions specifically during cytokinesis. However, the use of small peptides interfering with protein–protein interactions is emerging as a valid alternative pharmacological approach and peptides able to interfere with the interaction between INCENP and Aurora B have already been successfully used to impair CPC activity (61). Therefore, small peptides designed to impair CPC binding to its cytokinesis partners – such as KIF20A, CIT-K, and CHMP4 proteins – could be used to specifically inhibit this complex during cytokinesis and offer an alternative strategy for the development of highly targeted and potentially less toxic anticancer therapies.

## Concluding Remarks

Considerable progresses have been made in the last years to understand the multiple roles of Aurora B and the CPC during the rapid and highly coordinated process of cytokinesis. These studies have indicated that the CPC plays important functions in every step of this process, from the initial determination of the cleavage plane to the final abscission of the two daughter cells. They have also determined that the CPC controls the proper segregation of the genomic material not only in early mitosis by controlling kinetochore–microtubule attachments but also later in cytokinesis by delaying abscission in the presence of lagging chromosomes at the cleavage site. These findings suggest that the CPC probably deserves the appellative of “guardian of genome segregation” for its key role in preventing aneuploidy and CIN. However, important questions still remain open. Do Aurora B and other kinases, such as Plk1 and CIT-K, share common substrates during cytokinesis? How is Aurora B function coordinated with that of other kinases to cooperatively regulate the function of their various substrates during cytokinesis? Elucidating these mechanisms will keep scientist in the field busy for many years to come.

## Conflict of Interest Statement

The authors declare that the research was conducted in the absence of any commercial or financial relationships that could be construed as a potential conflict of interest.

## References

[1] Fujiwara T Bandi M Nitta M Ivanova EV Bronson RT Pellman D. Cytokinesis failure generating tetraploids promotes tumorigenesis in p53-null cells. Nature (2005) 437:1043–7. 10.1038/nature04217 16222300

[2] Iwanaga Y Chi YH Miyazato A Sheleg S Haller K Peloponese JM Jr Heterozygous deletion of mitotic arrest-deficient protein 1 (MAD1) increases the incidence of tumors in mice. Cancer Res (2007) 67:160–6. 10.1158/0008-5472.CAN-06-3326 17210695

[3] Li M Fang X Wei Z York JP Zhang P. Loss of spindle assembly checkpoint-mediated inhibition of Cdc20 promotes tumorigenesis in mice. J Cell Biol (2009) 185:983–94. 10.1083/jcb.200904020 19528295 PMC2711613

[4] Weaver BA Silk AD Montagna C Verdier-Pinard P Cleveland DW. Aneuploidy acts both oncogenically and as a tumor suppressor. Cancer Cell (2007) 11:25–36. 10.1016/j.ccr.2006.12.003 17189716

[5] Lens SM Voest EE Medema RH. Shared and separate functions of polo-like kinases and Aurora kinases in cancer. Nat Rev Cancer (2010) 10:825–41. 10.1038/nrc2964 21102634

[6] Carmena M Wheelock M Funabiki H Earnshaw WC. The chromosomal passenger complex (CPC): from easy rider to the godfather of mitosis. Nat Rev Mol Cell Biol (2012) 13:789–803. 10.1038/nrm3474 23175282 PMC3729939

[7] Gruneberg U Neef R Honda R Nigg EA Barr FA. Relocation of Aurora B from centromeres to the central spindle at the metaphase to anaphase transition requires MKlp2. J Cell Biol (2004) 166:167–72. 10.1083/jcb.200403084 15263015 PMC2172317

[8] van der Horst A Vromans MJ Bouwman K van der Waal MS Hadders MA Lens SM. Inter-domain cooperation in INCENP promotes Aurora B relocation from centromeres to microtubules. Cell Rep (2015) 12:380–7. 10.1016/j.celrep.2015.06.038 26166576

[9] Douglas ME Mishima M. Still entangled: assembly of the central spindle by multiple microtubule modulators. Semin Cell Dev Biol (2010) 21:899–908. 10.1016/j.semcdb.2010.08.005 20732438

[10] Mishima M Kaitna S Glotzer M. Central spindle assembly and cytokinesis require a kinesin-like protein/RhoGAP complex with microtubule bundling activity. Dev Cell (2002) 2:41–54. 10.1016/S1534-5807(01)00110-1 11782313

[11] Adams RR Tavares AA Salzberg A Bellen HJ Glover DM. *Pavarotti* encodes a kinesin-like protein required to organize the central spindle and contractile ring for cytokinesis. Genes Dev (1998) 12:1483–94. 10.1101/gad.12.10.1483 9585508 PMC316841

[12] Powers J Bossinger O Rose D Strome S Saxton W. A nematode kinesin required for cleavage furrow advancement. Curr Biol (1998) 8:1133–6. 10.1016/S0960-9822(98)70470-1 9778533 PMC3209536

[13] Raich WB Moran AN Rothman JH Hardin J. Cytokinesis and midzone microtubule organization in *Caenorhabditis elegans* require the kinesin-like protein ZEN-4. Mol Biol Cell (1998) 9:2037–49. 10.1091/mbc.9.8.2037 9693365 PMC25457

[14] Kamijo K Ohara N Abe M Uchimura T Hosoya H Lee JS Dissecting the role of Rho-mediated signaling in contractile ring formation. Mol Biol Cell (2006) 17:43–55. 10.1091/mbc.E05-06-0569 16236794 PMC1345645

[15] Somers WG Saint R. A RhoGEF and Rho family GTPase-activating protein complex links the contractile ring to cortical microtubules at the onset of cytokinesis. Dev Cell (2003) 4:29–39. 10.1016/S1534-5807(02)00402-1 12530961

[16] Yuce O Piekny A Glotzer M. An ECT2-centralspindlin complex regulates the localization and function of RhoA. J Cell Biol (2005) 170:571–82. 10.1083/jcb.200501097 16103226 PMC2171506

[17] Zhao WM Fang G. MgcRacGAP controls the assembly of the contractile ring and the initiation of cytokinesis. Proc Natl Acad Sci U S A (2005) 102:13158–63. 10.1073/pnas.0504145102 16129829 PMC1201590

[18] Lekomtsev S Su KC Pye VE Blight K Sundaramoorthy S Takaki T Centralspindlin links the mitotic spindle to the plasma membrane during cytokinesis. Nature (2012) 492:276–9. 10.1038/nature11773 23235882

[19] Hutterer A Glotzer M Mishima M. Clustering of centralspindlin is essential for its accumulation to the central spindle and the midbody. Curr Biol (2009) 19:2043–9. 10.1016/j.cub.2009.10.050 19962307 PMC3349232

[20] Douglas ME Davies T Joseph N Mishima M. Aurora B and 14-3-3 coordinately regulate clustering of centralspindlin during cytokinesis. Curr Biol (2010) 20:927–33. 10.1016/j.cub.2010.03.055 20451386 PMC3348768

[21] Bieling P I Telley A Surrey T. A minimal midzone protein module controls formation and length of antiparallel microtubule overlaps. Cell (2010) 142:420–32. 10.1016/j.cell.2010.06.033 20691901

[22] Kurasawa Y Earnshaw WC Mochizuki Y Dohmae N Todokoro K. Essential roles of KIF4 and its binding partner PRC1 in organized central spindle midzone formation. EMBO J (2004) 23:3237–48. 10.1038/sj.emboj.7600347 15297875 PMC514520

[23] Subramanian R Wilson-Kubalek EM Arthur CP Bick MJ Campbell EA Darst SA Insights into antiparallel microtubule crosslinking by PRC1, a conserved nonmotor microtubule binding protein. Cell (2010) 142:433–43. 10.1016/j.cell.2010.07.012 20691902 PMC2966277

[24] Zhu C Jiang W. Cell cycle-dependent translocation of PRC1 on the spindle by Kif4 is essential for midzone formation and cytokinesis. Proc Natl Acad Sci U S A (2005) 102:343–8. 10.1073/pnas.0408438102 15625105 PMC544298

[25] Zhu C Lau E Schwarzenbacher R Bossy-Wetzel E Jiang W. Spatiotemporal control of spindle midzone formation by PRC1 in human cells. Proc Natl Acad Sci U S A (2006) 103:6196–201. 10.1073/pnas.0506926103 16603632 PMC1458854

[26] Bastos RN Gandhi SR Baron RD Gruneberg U Nigg EA Barr FA. Aurora B suppresses microtubule dynamics and limits central spindle size by locally activating KIF4A. J Cell Biol (2013) 202:605–21. 10.1083/jcb.201301094 23940115 PMC3747307

[27] Bastos RN Cundell MJ Barr FA. KIF4A and PP2A-B56 form a spatially restricted feedback loop opposing Aurora B at the anaphase central spindle. J Cell Biol (2014) 207:683–93. 10.1083/jcb.201409129 25512391 PMC4274259

[28] Bassi ZI Audusseau M Riparbelli MG Callaini G D’Avino PP. Citron kinase controls a molecular network required for midbody formation in cytokinesis. Proc Natl Acad Sci U S A (2013) 110:9782–7. 10.1073/pnas.1301328110 23716662 PMC3683733

[29] Bassi ZI Verbrugghe KJ Capalbo L Gregory S Montembault E Glover DM Sticky/Citron kinase maintains proper RhoA localization at the cleavage site during cytokinesis. J Cell Biol (2011) 195:595–603. 10.1083/jcb.201105136 22084308 PMC3257531

[30] Gai M Camera P Dema A Bianchi F Berto G Scarpa E Citron kinase controls abscission through RhoA and anillin. Mol Biol Cell (2011) 22:3768–78. 10.1091/mbc.E10-12-0952 21849473 PMC3192857

[31] Gruneberg U Neef R Li X Chan EH Chalamalasetty RB Nigg EA KIF14 and citron kinase act together to promote efficient cytokinesis. J Cell Biol (2006) 172:363–72. 10.1083/jcb.200511061 16431929 PMC2063646

[32] Watanabe S De Zan T Ishizaki T Narumiya S. Citron kinase mediates transition from constriction to abscission through its coiled-coil domain. J Cell Sci (2013) 126:1773–84. 10.1242/jcs.116608 23444367

[33] Archambault V Glover DM. Polo-like kinases: conservation and divergence in their functions and regulation. Nat Rev Mol Cell Biol (2009) 10:265–75. 10.1038/nrm2653 19305416

[34] Carmena M Pinson X Platani M Salloum Z Xu Z Clark A The chromosomal passenger complex activates polo kinase at centromeres. PLoS Biol (2012) 10:e1001250. 10.1371/journal.pbio.1001250 22291575 PMC3265468

[35] Kachaner D Pinson X El Kadhi KB Normandin K Talje L Lavoie H Interdomain allosteric regulation of polo kinase by Aurora B and Map205 is required for cytokinesis. J Cell Biol (2014) 207:201–11. 10.1083/jcb.201408081 25332165 PMC4210448

[36] Earnshaw WC Cooke CA. Analysis of the distribution of the INCENPs throughout mitosis reveals the existence of a pathway of structural changes in the chromosomes during metaphase and early events in cleavage furrow formation. J Cell Sci (1991) 98(Pt 4):443–61. 1860899 10.1242/jcs.98.4.443

[37] Fuller BG Lampson MA Foley EA Rosasco-Nitcher S Le KV Tobelmann P Midzone activation of Aurora B in anaphase produces an intracellular phosphorylation gradient. Nature (2008) 453:1132–6. 10.1038/nature06923 18463638 PMC2724008

[38] Basant A Lekomtsev S Tse YC Zhang D Longhini KM Petronczki M Aurora B kinase promotes cytokinesis by inducing centralspindlin oligomers that associate with the plasma membrane. Dev Cell (2015) 33:204–15. 10.1016/j.devcel.2015.03.015 25898168 PMC4431772

[39] Steigemann P Wurzenberger C Schmitz MH Held M Guizetti J Maar S Aurora B-mediated abscission checkpoint protects against tetraploidization. Cell (2009) 136:473–84. 10.1016/j.cell.2008.12.020 19203582

[40] Capalbo L Montembault E Takeda T Bassi ZI Glover DM D’Avino PP. The chromosomal passenger complex controls the function of endosomal sorting complex required for transport-III Snf7 proteins during cytokinesis. Open Biol (2012) 2:120070. 10.1098/rsob.120070 22724069 PMC3376741

[41] Carlton JG Caballe A Agromayor M Kloc M Martin-Serrano J. ESCRT-III governs the Aurora B-mediated abscission checkpoint through CHMP4C. Science (2012) 336:220–5. 10.1126/science.1217180 22422861 PMC3998087

[42] McCullough J Colf LA Sundquist WI. Membrane fission reactions of the mammalian ESCRT pathway. Annu Rev Biochem (2013) 82:663–92. 10.1146/annurev-biochem-072909-101058 23527693 PMC4047973

[43] Wollert T Wunder C Lippincott-Schwartz J Hurley JH. Membrane scission by the ESCRT-III complex. Nature (2009) 458:172–7. 10.1038/nature07836 19234443 PMC2743992

[44] Carlton JG Martin-Serrano J. Parallels between cytokinesis and retroviral budding: a role for the ESCRT machinery. Science (2007) 316:1908–12. 10.1126/science.1143422 17556548

[45] Elia N Sougrat R Spurlin TA Hurley JH Lippincott-Schwartz J. Dynamics of endosomal sorting complex required for transport (ESCRT) machinery during cytokinesis and its role in abscission. Proc Natl Acad Sci U S A (2011) 108:4846–51. 10.1073/pnas.1102714108 21383202 PMC3064317

[46] Bischoff JR Anderson L Zhu Y Mossie K Ng L Souza B A homologue of *Drosophila* Aurora kinase is oncogenic and amplified in human colorectal cancers. EMBO J (1998) 17:3052–65. 10.1093/emboj/17.11.3052 9606188 PMC1170645

[47] Kanda A Kawai H Suto S Kitajima S Sato S Takata T Aurora-B/AIM-1 kinase activity is involved in Ras-mediated cell transformation. Oncogene (2005) 24:7266–72. 10.1038/sj.onc.1208884 16027732

[48] Zhou H Kuang J Zhong L Kuo WL Gray JW Sahin A Tumour amplified kinase STK15/BTAK induces centrosome amplification, aneuploidy and transformation. Nat Genet (1998) 20:189–93. 10.1038/2496 9771714

[49] Nguyen HG Makitalo M Yang D Chinnappan D St Hilaire C Ravid K. Deregulated Aurora-B induced tetraploidy promotes tumorigenesis. FASEB J (2009) 23:2741–8. 10.1096/fj.09-130963 19332642 PMC2717782

[50] Marxer M Foucar CE Man WY Chen Y Tang H Ma HT Tetraploidization increases sensitivity to Aurora B kinase inhibition. Cell Cycle (2012) 11(13):2567–77. 10.4161/cc.20947 22722494

[51] Chen YJ Chen CM Twu NF Yen MS Lai CR Wu HH Overexpression of Aurora B is associated with poor prognosis in epithelial ovarian cancer patients. Virchows Arch (2009) 455:431–40. 10.1007/s00428-009-0838-3 19838728

[52] Huang PY Li Y Luo DH Hou X Zeng TT Li MQ Expression of Aurora-B and FOXM1 predict poor survival in patients with nasopharyngeal carcinoma. Strahlenther Onkol (2015) 191:649–55. 10.1007/s00066-015-0840-4 25986250

[53] Tanaka S Arii S Yasen M Mogushi K Su NT Zhao C Aurora kinase B is a predictive factor for the aggressive recurrence of hepatocellular carcinoma after curative hepatectomy. Br J Surg (2008) 95:611–9. 10.1002/bjs.6011 18311747

[54] Xia R Chen S Chen Y Zhang W Zhu R Deng A. A chromosomal passenger complex protein signature model predicts poor prognosis for non-small-cell lung cancer. OncoTargets Ther (2015) 8:721–6. 10.2147/OTT.S81328 25897247 PMC4396580

[55] Zhang Y Jiang C Li H Lv F Li X Qian X Elevated Aurora B expression contributes to chemoresistance and poor prognosis in breast cancer. Int J Clin Exp Pathol (2015) 8:751–7. 25755770 PMC4348845

[56] Moore AS Blagg J Linardopoulos S Pearson AD. Aurora kinase inhibitors: novel small molecules with promising activity in acute myeloid and Philadelphia-positive leukemias. Leukemia (2010) 24:671–8. 10.1038/leu.2010.15 20147976

[57] Huang HC Shi J Orth JD Mitchison TJ. Evidence that mitotic exit is a better cancer therapeutic target than spindle assembly. Cancer Cell (2009) 16:347–58. 10.1016/j.ccr.2009.08.020 19800579 PMC2758291

[58] D’Avino PP Savoian MS Glover DM. Mutations in sticky lead to defective organization of the contractile ring during cytokinesis and are enhanced by Rho and suppressed by Rac. J Cell Biol (2004) 166:61–71. 10.1083/jcb.200402157 15240570 PMC2172139

[59] Ganem NJ Storchova Z Pellman D. Tetraploidy, aneuploidy and cancer. Curr Opin Genet Dev (2007) 17:157–62. 10.1016/j.gde.2007.02.011 17324569

[60] Storchova Z Pellman D. From polyploidy to aneuploidy, genome instability and cancer. Nat Rev Mol Cell Biol (2004) 5:45–54. 10.1038/nrm1276 14708009

[61] Gohard FH St-Cyr DJ Tyers M Earnshaw WC. Targeting the INCENP IN-box-Aurora B interaction to inhibit CPC activity in vivo. Open Biol (2014) 4:140163. 10.1098/rsob.140163 25392451 PMC4248066

